# Characterisation of Basic Sites on Ga_2_O_3_, MgO, and ZnO with Preadsorbed Ethanol and Ammonia—IR Study

**DOI:** 10.3390/molecules29133070

**Published:** 2024-06-27

**Authors:** Jerzy Podobiński, Jerzy Datka

**Affiliations:** Jerzy Haber Institute of Catalysis and Surface Chemistry, Polish Academy of Sciences, Niezapominajek 8, 30-239 Krakow, Poland; jerzy.podobinski@ikifp.edu.pl

**Keywords:** Ga_2_O_3_, MgO, ZnO basic sites, CO_2_ adsorption, ethoxy groups, ammonia adsorption

## Abstract

The effect of adsorption of ethanol and ammonia on the basicity of Ga_2_O_3_, MgO, and ZnO was examined via IR studies of CO_2_ adsorption. Ethanol reacts with OH groups on Ga_2_O_3_, and MgO, forming ethoxyl groups. The substitution of surface hydroxyls by ethoxyls increases the basicity of the neighbouring oxygen. The ethoxyl groups that also form on ZnO do not contain surface OH groups, but the mechanism of their formation is different. On ZnO, ethoxy groups are formed by the reaction of ethanol with surface oxygens. The presence of ethoxyls on ZnO decreases the basicity because some surface oxygens are already engaged in the bonding of ethoxyl groups. The effect of ammonia adsorption on basicity is different for each oxide. For Ga_2_O_3_, ammonia adsorption increases the basicity of neighbouring oxygen sites. Ammonia is not adsorbed on MgO; therefore, it does not change the basicity of this oxide. Ammonia adsorbed on ZnO forms coordination bonds with Zn sites; it does not change the number of basic sites but changes how carbonate species are bonded to surface sites.

## 1. Introduction

Basic catalysts play an important role in chemical industry. They catalyse various reactions of organic molecules such as hydrogenation, double-bond isomerization, dehydrocylcodimerisation, amination, aldol condensation, Michael addition, the nitroaldol reaction, the Tishchenko reaction, conjugate addition of alcohol, cyanoetylation, and others [1,2,3]. Therefore, the determination of basic properties of catalysts is a fundamental problem in science and technology. It is commonly accepted that CO_2_ is the right choice of probe molecule for basicity studies of solids [4,5,6,7,8,9,10,11,12,13,14,15,16,17,18,19]. In order to gain insight into the concentration and strength of basic sites, CO_2_ temperature-programmed desorption (TPD) techniques and IR spectroscopy are usually implemented. IR spectroscopy can be used to distinguish between two kinds of basic sites: OH^−^ and O^2−^. CO_2_ reacts with surface O^2−^, forming carbonate ions (CO_3_^2−^). It also reacts with basic OH^−^, forming bicarbonate ions (HCO_3_^−^). These species have different IR spectra, and there are bands characteristic of each of these forms [10,13,15,16,17]. A very good review on the IR spectra of CO_2_ adsorbed on various oxides was written by Busca and Lorenzelli [5].

Generally, all the oxygens and hydroxyls on the surface of metal oxides have negative charges. The lower is the electronegativity of the metal, the more negative the oxygens and hydroxyls. The local environment of surface sites may, however, cause some of them to be more negative than others. They may react with adsorbed CO_2_, forming carbonates (CO_3_^2−^) or bicarbonates (HCO_3_^−^). The oxygens and hydroxyls that are able to form CO_3_^2−^ or HCO_3_^−^ will be denoted as O^2−^ and OH^−^. 

We elaborated a new method to conduct quantitative IR studies of concentration of both O^2−^ and OH^−^ on oxides [18] and determined the concentrations of these sites on ZrO_2_, CeO_2_, Al_2_O_3_, Ga_2_O_3_, CuO, ZnO, and MgO [18,19]. In each case, the total concentration of basic sites (O^2−^ plus OH^−^), as determined with IR spectroscopy, was very close to the concentration of all the sites identified in desorption experiments.

We studied also the effect of coadsorption of ethanol and ammonia on the basicity of alumina [20]. It has been found [20] that ethanol reacts with surface hydroxyls, forming ethoxyl groups, and that the substitution of hydroxyls by ethoxyls increases the negative charge on neighbouring surface oxygen atoms, making them sufficiently basic to react with CO_2_ and form CO_3_^−^. The adsorbed ammonia interacts with triccordinated surface Al atoms, and the transfer of electrons to surface sites increases the basicity of neighbouring sites. 

The present study concerns the basic properties of Ga_2_O_3_, MgO, and ZnO with preadsorbed ethanol and ammonia. The results from ZnO seem to be specially interesting because this oxide, unlike Al_2_O_3_, Ga_2_O_3_ and MgO, does not contain surface hydroxyls. 

## 2. Results and Discussion

OH groups on Ga_2_O_3_, MgO, and ZnO

**Figure 1:**
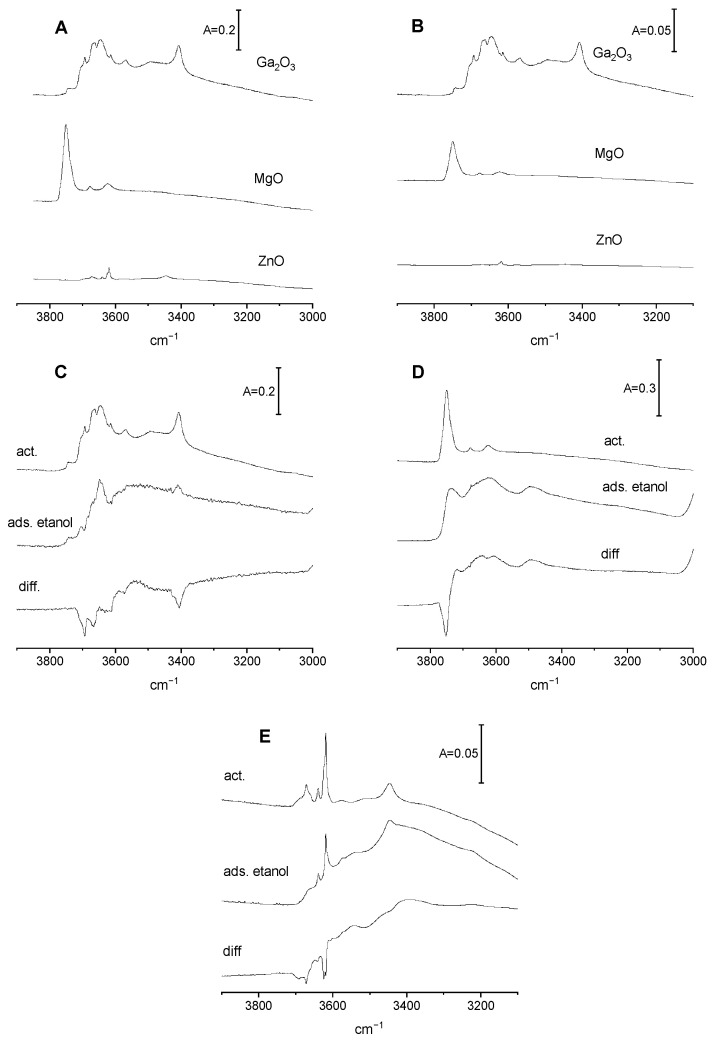
(**A**,**B**)—The spectra of OH groups on Ga_2_O_3_, MgO, and ZnO normalized to sample mass (**A**) and to surface area (**B**). (**C**–**E**)—OH groups on Ga_2_O_3_ (**C**), MgO, (**D**) and ZnO (**E**) activated upon reaction with ethanol and difference spectra.

The spectra of OH groups on Ga_2_O_3_, MgO, and ZnO activated at 720 K are presented in Figure 1A,B. The spectra presented in Figure 1A are normalized to the mass, and those presented in Figure 1B are normalized to the surface area of oxides. 

### 2.1. OH Groups on Ga_2_O_3_

The IR spectrum of OH groups on the surface of Ga_2_O_3_ (Figure 1A) shows several maxima. The multiplicity of these OH bands may be related to the fact that several polymorphs of gallia (denoted as α, β and γ—Ref. [16]) exist. Moreover, OH groups may be bonded to a gallium atom that is tetra-coordinated, a gallium atom that is hexacoordinated, or to both of them. The interpretation of our spectrum of OH groups on gallia was based on the interpretations given in previous work by Collins et al. [16], as well as Otero Arean and Lavalley’ groups [21,22,23,24]. The weak band at 3743 cm^−1^ may be related to Ga-OH, in which Ga is hexacoordinated; the strong band at 3692 cm^−1^ may be related to Ga-OH, in which Ga is tetra-coordinated; and the bands at 3670–3662 cm^−1^ may be related to OH interacting with two hexacoordinated Ga. The band at 3644 cm^−1^ may be attributed to OH interacting with both tetra-coordinated and hexacoordinated Ga. According to Collins et al. [16], these Ga-OH groups differ in net electrical charge. The hydroxyls vibrating at 3743, 3692, 3670–3362, and 3644 cm^−1^ have the charges −0.5, −0.25, 0, and +0.25, respectively. It should be noted that Knozinger and Ratnasamy [25] reported the same correlation for hydroxyls on an alumina surface: Al-OH at higher stretching frequencies showed a more negative charge. 

### 2.2. OH Groups on MgO 

The spectrum of OH groups on the MgO surface (Figure 1A) shows a distinct band at 3750 cm^−1^. This band is asymmetric and shows a shoulder at around 3730 cm^−1^. We also observed weaker bands at 3620 and 3680 cm^−1^. The OH groups on MgO have already been the subject of studies by IR spectroscopy, as well as by periodic (VASP) and cluster DFT simulation [26,27]. Several models were considered, and the effect of location of hydroxyls and their coordination were discussed. Both free and hydrogen-bonded hydroxyls are present. Both Knozinger et al. [26] and Chizallet et al. [27] identified four types of OH groups on the MgO surface. They were Mg-OH, Mg-OH-Mg (denoted as types A and B, respectively), and two kinds of hydrogen-bonded hydroxyls (types C and D). It seems possible that the band at 3750 cm^−1^ band observed in our study (Figure 1A) may be attributed to OH type A and that the shoulder at ca. 3730 cm^−1^ may be attributed to OH type B. We suppose also that weak bands at 3620 and 3680 cm^−1^ are due to hydrogen-bonded hydroxyls (types C and D). Our earlier study [19] showed that dehydroxylation at a high temperature (900 K) removed Mg-OH (type A) in the first order, indicating that these hydroxyls are less stable at high temperatures. 

### 2.3. OH Groups on ZnO 

The spectra of OH groups on the ZnO surface are presented in Figure 1A,B. The intensity of OH bands is very low compared with those of Ga_2_O_3_ and MgO. This is best seen by comparing OH intensities normalized to the same surface area (Figure 1B). The interpretation of the spectra of OH groups was based on earlier results from Noei et al. [28], who studied the single-crystal surfaces of ZnO nanoparticles. A weak band at 3620 cm^−1^ was attributed to OH groups on the ZnO (0001) surface formed by the dissociation of water on oxygen vacancy sites. Another very weak band at 3640 cm^−1^ was attributed to OH on a mixed-terminated ZnO (1010) surface. 

Reaction of ethanol with OH groups on Ga_2_O_3_, MgO, and ZnO

The reaction with ethanol consumed some Ga-OH groups and produced ethoxy groups. The data presented in Figure 1C evidence that only the hydroxyls with the highest stretching frequencies i.e., these that have a net negative charge or are neutral, react with ethanol. The reaction of methanol with surface sites on oxides was studied by Bianchi et al. [29]. These authors considered various mechanisms by which these reactions might produce methoxy groups. One of these mechanisms involved the condensation of alcohol with surface hydroxyls, forming alkoxy groups and water according to the following scheme:

Me-OH + HO-R = Me-OR + H_2_O (1)

**Figure 2:**
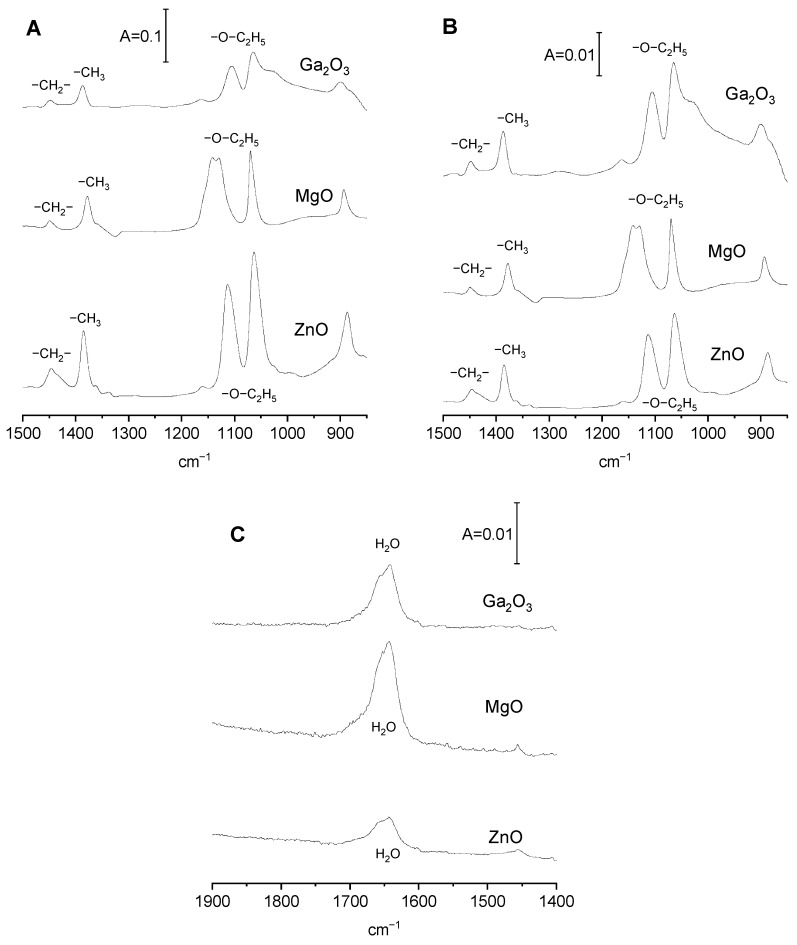
(**A**,**B**)—The spectra of ethoxy groups on Ga_2_O_3_, MgO, and ZnO normalized to sample mass (**A**) and to surface area (**B**). (**C**)—the spectra of the product of reaction of ethanol with Ga_2_O_3_, MgO, and ZnO. The reaction products were adsorbed on dehydrated zeolite NaY.

According to this mechanism, the formation of ethoxy groups was accompanied by the production of water. In order to check whether water is indeed produced by the reaction of ethanol with surface hydroxyl groups, we adsorbed the dose of ethanol (0.5 µmol/100 mg of Ga_2_O_3_) on the wafer of gallia at room temperature. Subsequently, the gallia wafer with ethanol was heated to 370 K and the product of the reaction was desorbed into a cold trap. The molecules stored in the cold trap were next adsorbed at room temperature on a wafer of zeolite NaY (activated at vacuum at 670 K). The spectrum of molecules that were the product of the reaction of ethanol on gallia adsorbed on zeolite NaY is presented in Figure 2C (top spectrum). This spectrum shows a band at 1640 cm^−1^, which represents the deformation vibrations of a water molecule, evidencing that water is indeed formed in the reaction of ethanol with surface hydroxyls on gallia according to the mechanism presented above. 

The spectrum of ethoxy groups formed in gallia is presented in Figure 2B (top spectrum). The spectrum shows the bands at 1385 cm^−1^ that represent CH_3_ deformation, as well as three bands typical of ethoxy groups: bands at 895 and 1063 cm^−1^ representing symmetric and asymmetric C-C-O entity and a 1100 cm^−1^ band that is interpreted [30] as a combination of two vibrations: deformation (δ) of Ga-O-C and rocking (r) of CH_3_ group. The band at 1063 cm^−1^ is complex—it comprises two submaxima. The profound analysis of two other bands of ethoxy groups on gallia described in our previous study [19] showed that both 895 and 1100 cm^−1^ bands composed of two submaxima too. Therefore, all the three bands of ethoxy groups on gallia comprise two components, showing that two kinds of ethoxy groups were formed. They may be monodentate and bidentate ethoxyls interacting with one and two gallium atoms, respectively. It is possible that monodentate and bidentate ethoxyls are formed by the interaction of ethanol with OH groups interacting with one and two Ga, respectively. 

Reaction of ethanol with MgO consumes primarily all the Mg-OH groups (Figure 1D) and produces ethoxyls. The experiment in which the product of the reaction of ethanol on MgO was trapped in cold trap and subsequently adsorbed on zeolite NaY (according to the procedure described above) showed, that water was also the product of this reaction according to scheme (1). The spectrum of water being the product of reaction adsorbed on zeolite NaY is presented in Figure 2A. 

The spectrum of ethoxy groups formed in MgO is presented in Figure 2B. The spectrum shows the bands at 1379 and 1450 cm^−1^ of CH_3_ and CH_2_ deformation, as well as three bands typical of ethoxy groups: bands at 894 and 1070 cm^−1^ typical of symmetric and asymmetric vibrations of a C-C-O entity and the doublet at 1129 and 1162 cm^−1^, which is interpreted [30] as combination of two vibrations: deformation (δ) of Ga-O-C and rocking (r) of a CH_3_ group. 

The results concerning the reaction of ethanol with surface sites on ZnO will now be discussed. The result for ZnO was found to be different from that observed for both Ga_2_O_3_ and MgO, because ZnO, unlike Ga_2_O_3_ and MgO, contains a very low concentration of surface hydroxyls. This is seen in Figure 1B. Therefore, it was expected that ethoxy groups would be not formed by the reaction of ethanol on ZnO. However, a significant amount of ethoxyls was formed on ZnO. This is best seen in Figure 2B, in which the spectra of ethoxy groups are normalized to the surface area of oxides. The intensities of the bands of ethoxy groups on ZnO are comparable to those found for Ga_2_O_3_ and MgO, even though ZnO does not contain a significant number of surface hydroxyl groups (it was assumed that the extinction coefficients of the bands are comparable). This evidences that the mechanism of the formation of ethoxyl groups is different as a result of the mechanism (1) according to which the surface hydroxyls are engaged. As mentioned, Bianchi et al. [29] considered several mechanisms for the reaction of alcohols with oxides. One of these mechanisms assumes the reaction of alcohol with Me-O-Me entities, as follows:

Me-O-Me + HO-R = Me-OH + Me-O-R(2)

This mechanism postulates the formation of new hydroxyl groups. It should be mentioned that according to our earlier results [31], the reaction of ethanol with ceria produced such new hydroxyls. In the present study (Figure 1E), no new hydroxyls were formed upon the reaction of ethanol on ZnO; therefore, the scheme (2) does not describe this reaction. Moreover, water was formed in this reaction, as shown by the presence of a 1640 cm^−1^ band in the spectrum of reaction products trapped in the cold trap and readsorbed on zeolite NaY (Figure 2C). 

Bianchi et al. [29] proposed also another mechanism of reaction of alcohols with surface sites on oxides:

Me-O-Me + 2 HO-R = 2 Me-OR + H_2_O (3)

According to this mechanism, the reaction of alcohols neither consumes nor creates new surface hydroxyls. Ethoxy groups and water are formed. However, the amount of water formed (one water molecule per two alkoxy group) is only a half of the amount of water that would be formed if reaction occurred according to mechanism (1) (one water molecule per one ethoxy group). According to the data presented in Figure 2C, the intensity of the band of water produced on ZnO is ca 35–65% of the intensity of the water band produced on Ga_2_O_3_ and MgO (the amount of adsorbed ethanol was the same in all these three cases). This fact, together with the fact that no new hydroxyls are formed by the reaction of ethanol on ZnO, suggests that this reaction occurs according to mechanism (3). It should be remembered that the reaction of ethanol with surface sites on Ga_2_O_3_ and MgO occurs according to mechanism (1).

In summary, it can be said that the reaction of ethanol with surface sites on Ga_2_O_3_ and MgO consumes basic hydroxyl groups, replacing then with ethoxyls. On the other hand, the reaction of ethanol with ZnO (not containing surface hydroxyls) replaces some surface oxygens with ethoxy groups. In this study, we will examine how these modifications influence the basic properties of oxides.

Interaction of ammonia with Ga_2_O_3_, MgO, and ZnO

**Figure 3:**
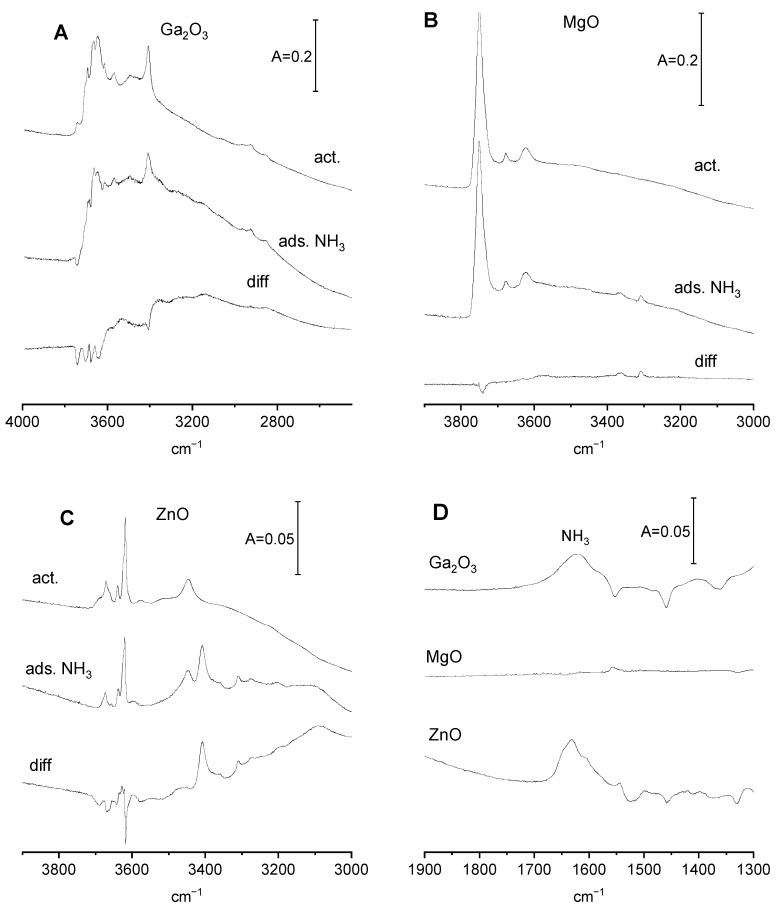
(**A**–**C**)—OH groups on Ga_2_O_3_ (**A**), MgO (**B**) and ZnO (**C**) with and without adsorbed ammonia, followed by desorption at 340 K, as well as difference spectra. (**D**)—the spectra of ammonia adsorbed on Ga_2_O_3_, MgO, and ZnO.

The spectra of OH groups on Ga_2_O_3_, MgO, and ZnO that interact with ammonia are presented in Figure 3A–C, and the spectra of ammonia that interact with the oxides are presented in Figure 3D. The adsorption of ammonia causes a decrease in the intensity of the OH bands of all the oxides, but ammonium ions are not produced (the band typical of ammonium ions at ca. 1450 cm^−1^ is absent—Figure 3D). It is possible that the decrease in bands of free OH may be due to the formation of hydrogen bonds. 

The spectra of ammonia adsorbed on Ga_2_O_3_ and ZnO (Figure 3D) show the band of ammonia interacting with electroacceptor sites (1620 cm^−1^ for Ga_2_O_3_ and 1630 cm^−1^ for ZnO). This band is absent for MgO. It may be supposed that these electroacceptor sites on Ga_2_O_3_ are surface tricoordinated gallium atoms, which (as for Al_2_O_3_) play the role of Lewis acid sites. For ZnO electroacceptor sites, the bonding ammonia may interact with Zn^2+^, which is an element known for forming numerous coordination compounds (engaging empty p and d orbitals) with a coordination number of four. An ammonia molecule is one of the most important ligands in zinc complexes, acting as electron donor to Zn^2+^. 

The effect of adsorption of ammonia on the basic properties of Ga_2_O_3_, MgO, and ZnO will be studied via the adsorption of CO_2_. 

CO_2_ adsorption on Ga_2_O_3_, MgO, and ZnO without preadsorbed ethanol and ammonia

**Figure 4:**
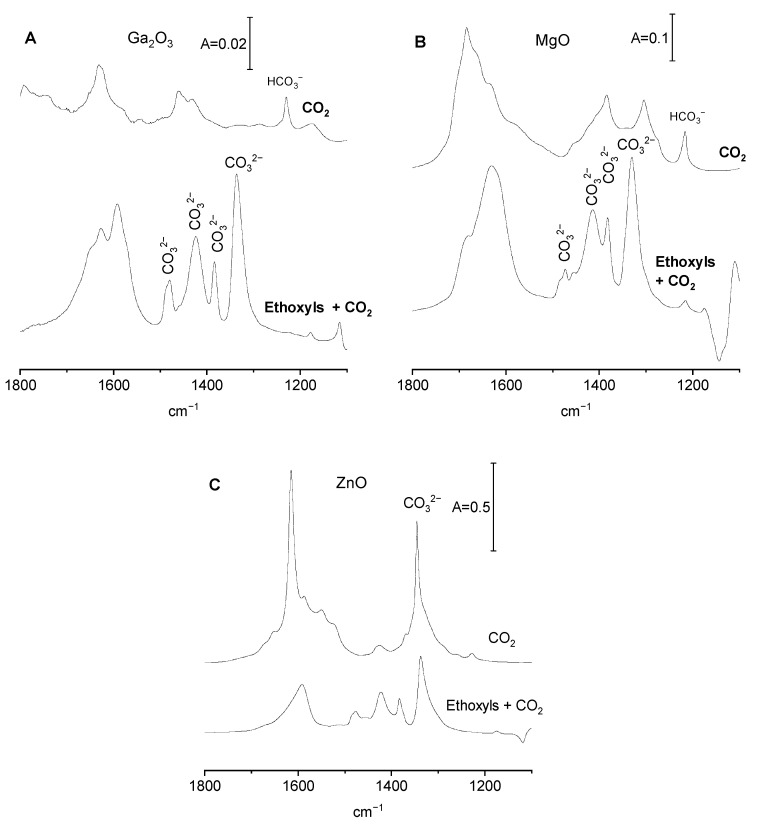
The spectra of CO_2_ adsorbed on Ga_2_O_3_, MgO, and ZnO, with and without ethoxyl groups.

The spectra of CO_2_ adsorbed on Ga_2_O_3_, MgO, and ZnO without preadsorbed ethanol and ammonia are presented in Figure 4A–C (top spectra). The adsorption of CO_2_ on these oxides has already been studied, and the results were described in our previous paper [19]. The most important conclusions will be revisited below.

CO_2_ adsorbed on Ga_2_O_3_ forms only bicarbonate species, indicating that OH^−^ sites are the only basic sites able to react with CO_2_. Both monodentate and bidentate bicarbonates were formed. Carbonate ions were not produced. Similar results were obtained in our previous study concerning alumina [20], as well as in a study by other authors [16] The analysis of the spectra in OH region showed that only ca. 10% of hydroxyls were sufficiently basic to react with CO_2_. The OH groups reacting with CO_2_ were the hydroxyls with the highest stretching frequencies, i.e., hydroxyls that were also reacting with ethanol. 

The adsorption of CO_2_ on MgO produces both bicarbonate and carbonate species, evidencing that the basic sites can be either OH^−^ and O^2−^. Both monodentate and bidentate bicarbonates were formed. Similar results were also obtained by other authors [32,33] The reaction with CO_2_ consumed ca. 10–20% of all the hydroxyls. The analysis of the OH spectra proved that the high-frequency Mg-OH terminal groups (type A) were more basic than Mg-OH-Mg (type B). 

ZnO contained primarily O^2−^ sites, and predominantly carbonate ions were formed by the adsorption of CO_2_, with the concentration of bicarbonates being very low. This is consistent with the fact that the intensity of OH bands is also very low (Figure 1B). 

Quantitative studies of the concentration of basic sites O^2−^ and OH^−^ ware also carried out. The extinction coefficients of diagnostic bands at ca. 1220 cm^−1^ 1440 cm^−1^ for HCO_3_^−^ and 1340 cm^−1^ for CO_3_^−^ were determined by the adsorption of measured doses of CO_2_ on ZrO_2_ and CeO_2_ [18], and subsequently, the concentrations of basic sites (separately, O^2−^ and OH^−^) were determined for ZrO_2_, CeO_2_, CuO, Al_2_O_3_, ZnO, Ga_2_O_3_, and MgO [19]. Moreover, the total concentration of all the basic sites (O^2−^ plus OH^−^) was determined in CO_2_-desorption experiments in which the amount of desorbed CO_2_ was monitored by IR spectroscopy. For all the mentioned oxides, the basicity measured by two independent methods (from the intensities of bicarbonate and carbonate bands and from desorption experiments) were primarily the same. 

The total concentration of basic sites on Ga_2_O_3_, MgO, and ZnO, both with and without CO₂ desorption as monitored by IR studies, is presented in Table 1. 

CO_2_ adsorption on Ga_2_O_3_, MgO, and ZnO with preadsorbed ethanol

The spectrum of bicarbonate and carbonate species formed by the reaction of CO_2_ with basic sites on gallia with preadsorbed ethanol (i.e., containing surface ethoxy groups) is presented in Figure 4A (bottom spectrum) and may be compared with the spectrum of bicarbonates formed on gallia without ethoxy groups (top spectrum). 

The band of bending of OH (δ_OH_ at 1233 cm^−1^) of bicarbonate species that is present in the spectrum of CO_2_ adsorbed on Ga_2_O_3_ without ethoxyls is nearly absent in the presence of ethoxyls. This may be explained by the fact that most basic Ga-OH were consumed by the reaction with ethanol and substituted by ethoxy groups. Bicarbonates were therefore not formed. The spectra of carbonate species formed on gallia with preadsorbed ethanol (Figure 4A) show some new bands that were absent when no ethanol was introduced. It may be supposed that the new bands at 1337 and 1592 cm^−1^ may be attributed to ν_sym CO3_ and ν_asym CO3_ in bidendate carbonates, respectively. The band at 1384 cm^−1^ may be assigned to monodentate carbonates, and the bands at 1425 and 1492 cm^−1^ may be assigned to polydentate carbonates. The presence of relatively strong bands of carbonate species formed in the presence of ethoxyl groups evidences that some surface oxygens that were not basic enough to react with CO_2_ became basic if Ga-OH were substituted by Ga-O-C_2_H_5_. Similar results (decrease in the contribution of basic OH^−^, increase in the basicity of oxygens) were obtained in our earlier studies of alumina with preadsorbed ethanol [20]. 

The spectra of CO_2_ adsorbed on MgO with and without preadsorbed ethanol are presented in Figure 4B. In the presence of ethoxy groups, the intensity of the 1216 cm^−1^ band of bicarbonates is lower than its intensity without ethoxyls. This result may be explained by the substitution of some Mg-OH by Mg-O-C_2_H_5_, decreasing the amount of hydroxyls able to react with CO_2_, forming bicarbonates. The data presented in Figure 4B contain new intense bands of carbonates, which were absent when no ethoxyls were present. The intense bands at 1331 and 1630 cm^−1^ may be assigned to bidentate carbonyls. The band at 1382 cm^−1^ may be due to monodentate carbonyls, and the bands at 1415 and 1473 cm^−1^ may be attributed to polydentate carbonyls. 

The behaviour of ZnO is different from that described above for Ga_2_O_3_ and MgO. ZnO (unlike Ga_2_O_3_ and MgO) primarily does not contain surface OH groups. While on Ga_2_O_3_ and MgO, ethoxy groups were formed by substitution of OH by O-C_2_H_5_ and ethoxyls on ZnO were formed on surface oxygens. The spectra of CO_2_ adsorbed on ZnO with and without ethoxy groups are presented in Figure 4C. Without ethoxy groups, bidentate carbonates (the bands at 1345 and 1615 cm^−1^) were nearly the only groups formed, whereas the contribution of bicarbonates was very weak. The spectrum in Figure 4C suggests that two kinds of bidentate carbonates are present. One kind (denoted as type A) is characterized by bands at 1345 and 1615 cm^−1^), while the second kind (type B) is characterized by shoulders at 1330 cm^−1^ and 1590 cm^−1^.

In the presence of ethoxy groups, the concentration of carbonates on ZnO is lower than without ethoxyls (contrary to Ga_2_O_3_ and MgO). This may be explained by the fact that on ZnO, ethoxy groups are formed on oxygen atoms; therefore, the amount of basic oxygens able to react with CO_2_ and form carbonates is lower. The analysis of the spectra presented in Figure 4C suggests that in the presence of ethoxy groups, the basic oxygen atoms responsible for the formation of bidentate carbonates of type A are blocked by ethoxyls. The basic oxygens responsible for the formation of carbonates type B remain. The band at 1383 cm^−1^ may be assigned to monodentate, and those at 1420 and 1480 cm^−1^ may be assigned to polydentate carbonates. 

The information on the concentration of all the basic sites (O^2−^ plus OH^−^) on the surfaces of all the three oxides with and without the ethoxy groups was obtained in the studies of desorption of CO_2_ monitored by IR according to the procedure described in our previous paper [19]. These concentrations are presented in Table 1. For Ga_2_O_3_ and MgO, the formation of ethoxy groups increased the basicity of oxides. This result agrees with the conclusions obtained from the analysis of the data presented in Figure 4A,B (increase in the intensity of carbonate bands). On the other hand, for ZnO, the formation of ethoxy groups decreased the basicity, a result that also agrees with the data presented in Figure 4C (decrease of carbonate bands). 

CO_2_ adsorption on Ga_2_O_3_, MgO, and ZnO with preadsorbed ammonia.

**Figure 5:**
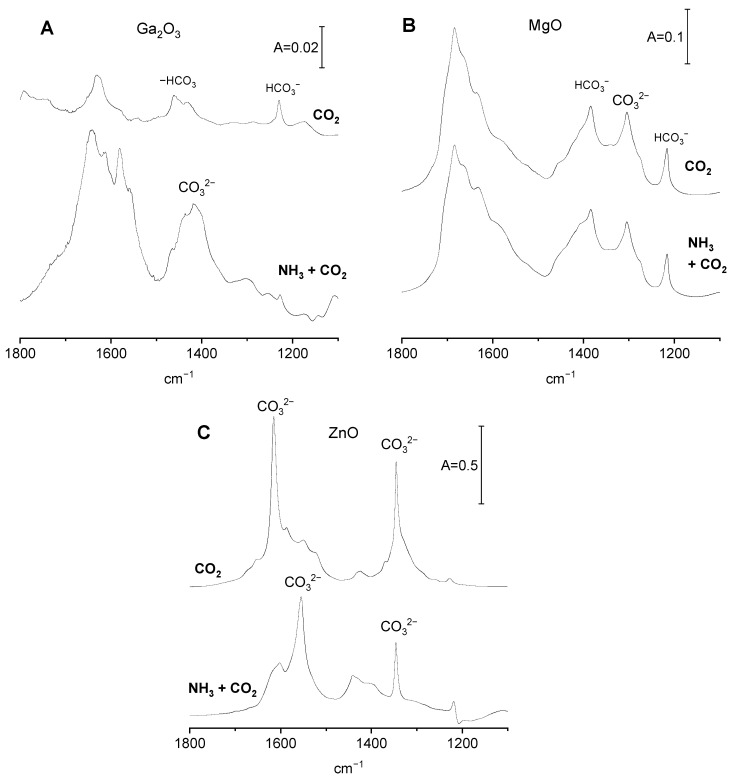
The spectra of CO_2_ adsorbed on Ga_2_O_3_, MgO, and ZnO with and without preadsorbed ammonia.

The spectra of CO_2_ adsorbed on Ga_2_O_3_ with and without preadsorbed ammonia are presented in Figure 5A. Ga_2_O_3_ contains only basic OH^−^; therefore, the bicarbonates are the only species formed upon CO_2_ adsorption. The amount of OH^−^ decreased when CO_2_ was adsorbed on gallia with preadsorbed ammonia. This is proven by the decrease in the intensity of band 1231 cm^−1^ of bicarbonate species. It may be explained by the engagement of surface hydroxyls in hydrogen bonding with ammonia (Figure 3A); therefore, the number of Ga-OH able to react with CO_2_ is smaller. In the presence of preadsorbed ammonia, the adsorption of CO_2_ produces significant amounts of polydentate carbonate ions (bands at 1416 and 1580 cm^−1^). 

According to the data presented in Figure 3B, ammonia is not adsorbed on MgO; therefore, the spectrum of CO_2_ adsorbed on MgO that made contact with ammonia is the same as the spectrum of CO_2_ adsorbed on MgO without ammonia (Figure 5B), evidencing that ammonia does not change the basic properties of MgO. 

The spectra of CO_2_ adsorbed on ZnO with and without preadsorbed ammonia are presented in Figure 5C. In the presence of preadsorbed ammonia, the bands of carbonates are present at somewhat different positions than without ammonia, suggesting that the presence of ammonia interacting with Zn^2+^ changes the manner of bonding of carbonate species to adsorption sites. The band of ν_sym CO3_ of bidendate is lower, and new bands at 1400 and 1440 cm^−1^ appear. These new bands may be assigned to monodentate and polydentate carbonates. The shift of the band of ν_asym CO3_ band from 1615 to 1555 cm^−1^ is also observed. 

Total concentration of all the basic sites

The information on the total concentration of all the basic sites (O^2−^ plus OH^−^) was obtained in the experiments on the desorption of CO_2_ monitored by IR spectroscopy according to the procedure described in our previous publication [19]. The concentration values for oxides with and without preadsorbed ethanol and ammonia are presented in Table 1. 

The concentrations were determined according to the procedure described in the Materials and Methods section.

The formation of ethoxy groups on Ga_2_O_3_ and MgO increases the total basicity, as observed before for alumina [20]. This may be explained by the substitution of surface hydroxyls by ethoxyls, which increases the negative charge on neighbouring oxygen atoms. The data presented in Figure 4A,B evidence the significant increase in the intensity of bands of carbonate species and the decrease in the intensity of bicarbonate bands, indicating a significant increase in the concentration of O^2−^ and decrease in the concentration of OH^−^. An opposite effect was observed for ZnO. The total concentration of all the basic sites decreases for ZnO with preadsorbed ethanol. A similar conclusion can be drawn from the analysis of the data presented in Figure 4C, in which the decrease in the intensity of carbonate bands is seen. This may be explained by a different mechanism of ethoxy-group formation on ZnO than on Ga_2_O_3_ and MgO. On ZnO, which does not contain surface hydroxyls, ethoxyl groups are formed on surface oxygens; therefore, the smaller number of oxygens may react with CO_2_, forming carbonate species. 

The adsorption of NH_3_ on Ga_2_O_3_ increases the concentration of basic sites (Table 1), a result that agrees with the conclusion drawn by comparing the spectra presented in Figure 5A (increase of intensity of carbonate bands). Similar effects were obtained for alumina [20]. This may be explained by transmission of electrons from ammonia molecule to adsorption sites (surface Ga atoms) and to neighbouring surface oxygens, which become sufficiently basic to react with CO_2_. Unlike Ga_2_O_3,_ MgO does not adsorb ammonia (no ammonia bands are seen in Figure 3D). There is no increase in the concentration of basic sites in the sample of MgO in contact with NH_3_ (Table 1) and no increase in the intensity of carbonate bands (Figure 5B). According to the data presented in Table 1, the adsorption of ammonia on ZnO does not change the number of basic sites. However, it changes how the carbonate species are bonded to surface sites (Figure 5C). 

## 3. Materials and Methods

Ga_2_O_3_ (ACS reagent, purity > 99.99%), MgO (POCH, Gliwice, Poland, analytical grade), ZnO (Aldrich 544906-10G Sigma-Aldrich, St. Louis, MO, USA nanopowder), and CO_2_ (Linde, Cracow, Poland, purity 99.98) were used. The surface areas (BET) of Ga_2_O_3_, MgO, and ZnO were 4, 8 and 12 m^2^/g, respectively.

For IR studies, all oxides were pressed into thin wafers of ca. 100–250 mg. Prior to IR experiments, wafers were evacuated in situ in an IR cell at 720 K for 30 min. (this temperature was optimal because at lower temperatures, atmospheric water and CO_2_ may not be removed completely; if the temperature is too high, partial dehydroxylation can occur). CO_2_, ethanol and ammonia were adsorbed at room temperature. For CO_2_ adsorption, the gas pressure in the IR cell was ca. 5 Torr. The band of molecular CO_2_ was present in the IR spectra, evidencing that all basic sites reacting with CO_2_ were saturated. For ethanol adsorption, the pressure in IR cell was ca. 3 Torr. Ethanol was in contact with oxide wafers for 3 min, and was subsequently desorbed by a 30 min evacuation at 370 K. In the experiments on ammonia adsorption, the pressure in the IR cell was 1–2 Torr. Ammonia was in contact with the oxide wafer for 3 min, and, subsequently, it was desorbed by evacuation at 340 K. In all experiments, the CO_2_ was adsorbed at room temperature on the oxide wafer with and without preadsorbed ethanol and ammonia. The method of determination of total basicity (the concentration of O^2−^ plus OH^−^) was described in detail in our previous paper [19]; some information on this method will be presented below. All the basic sites on alumina, with and without preadsorbed molecules, were first saturated with CO_2_ at room temperature. Next, the gaseous CO_2_ was removed from the cell and physiosorbed molecules were removed by a 1 min evacuation at room temperature. CO_2_ bonded to basic sites was subsequently desorbed at 470 K from the sample and trapped in a cold trap. The molecules trapped in the cold trap were next adsorbed on the wafer of zeolite NaY (which is a very efficient adsorbent). The amount of CO_2_ adsorbed on NaY was calculated from the intensity of the CO_2_ band at ca 2300 cm^−1^ and the extinction coefficient of this band. The total amount of CO_2_ adsorbed on basic sites was calculated by taking into account the amount of CO_2_ adsorbed on NaY, as well as the integrated intensities of all IR bands of the adsorbed (bi)carbonate species before and after evacuation at room temperature and after the desorption at 470 K. More details are given in [19].

IR spectra were recorded with a NICOLET 6700 spectrometer (Thermo Scientific, Cambridge, MA, USA) with a spectral resolution of 1 cm^−1^. The spectra were recorded in transmission mode.

## 4. Conclusions

The adsorption of ethanol on Ga_2_O_3_, MgO, and ZnO produces ethoxy groups. On Ga_2_O_3_ and MgO, ethoxyls are formed by the reaction with surface hydroxyls; on ZnO (which does not contain hydroxyls), they are formed by the reaction with surface oxygens. For Ga_2_O_3_ and MgO, the substitution of hydroxyls by ethoxyls increases the negative charge of neighbouring oxygen atoms, so they became sufficiently basic to react with adsorbed CO_2_, forming the carbonate species CO_3_^2−^. These carbonates were found to be mostly monodentate, bidentate, and polydentate species. For ZnO, some surface oxygens are engaged in the bonding of ethoxy groups. The number of O^2−^ basic sites able to react with CO_2_ is lower; therefore, less carbonate is formed than without ethoxyls. Ammonia adsorbed on Ga_2_O_3_ transfers electrons to adsorption sites, which increases the basicity of neighbouring oxygens. Ammonia is not adsorbed on MgO and does not change its basicity. The interaction of Zn sites with ammonia does not change the basicity of ZnO, but it does change how the carbonate species are bonded to surface sites.

## Figures and Tables

**Table 1:** Concentration of all the basic sites (O^2−^ plus OH^−^) on Ga_2_O_3_, MgO, and ZnO with and without preadsorbed ethanol and ammonia.

		Concentr. µmol/g
Ga_2_O_3_	None	5
	Ethoxyls	7
	NH_3_	9
MgO	None	19
	Ethoxyls	30
	NH_3_	19
ZnO	None	40
	Ethoxyls	21
	NH_3_	35

## Data Availability

The inquiries can be directed to corresponding author.
